# Integrated Informatics Analysis of Cancer-Related Variants

**DOI:** 10.1200/CCI.19.00132

**Published:** 2020-03-30

**Authors:** Kymberleigh A. Pagel, Rick Kim, Kyle Moad, Ben Busby, Lily Zheng, Collin Tokheim, Michael Ryan, Rachel Karchin

**Affiliations:** ^1^ The Institute for Computational Medicine, The Johns Hopkins University, Baltimore, MD; ^2^ In Silico Solutions, Falls Church, VA; ^3^ Mountain Genomics, Pittsburgh, PA; ^4^ Institute of Genetic Medicine, The Johns Hopkins University School of Medicine, Baltimore, MD; ^5^ Dana-Farber Cancer Institute, Boston, MA; ^6^ Departments of Biomedical Engineering, Oncology, and Computer Science, The Johns Hopkins University, Baltimore, MD

## Abstract

**PURPOSE:**

The modern researcher is confronted with hundreds of published methods to interpret genetic variants. There are databases of genes and variants, phenotype-genotype relationships, algorithms that score and rank genes, and in silico variant effect prediction tools. Because variant prioritization is a multifactorial problem, a welcome development in the field has been the emergence of decision support frameworks, which make it easier to integrate multiple resources in an interactive environment. Current decision support frameworks are typically limited by closed proprietary architectures, access to a restricted set of tools, lack of customizability, Web dependencies that expose protected data, or limited scalability.

**METHODS:**

We present the Open Custom Ranked Analysis of Variants Toolkit^1^ (OpenCRAVAT) a new open-source, scalable decision support system for variant and gene prioritization. We have designed the resource catalog to be open and modular to maximize community and developer involvement, and as a result, the catalog is being actively developed and growing every month. Resources made available via the store are well suited for analysis of cancer, as well as Mendelian and complex diseases.

**RESULTS:**

OpenCRAVAT offers both command-line utility and dynamic graphical user interface, allowing users to install with a single command, easily download tools from an extensive resource catalog, create customized pipelines, and explore results in a richly detailed viewing environment. We present several case studies to illustrate the design of custom workflows to prioritize genes and variants.

**CONCLUSION:**

OpenCRAVAT is distinguished from similar tools by its capabilities to access and integrate an unprecedented amount of diverse data resources and computational prediction methods, which span germline, somatic, common, rare, coding, and noncoding variants.

<<Instruction to Composition: SPECIAL SERIES: INFORMATICS TOOLS FOR CANCER RESEARCH AND CARE>>

## INTRODUCTION

Next-generation sequencing technologies have greatly reduced the cost of genome sequencing, increasing the availability of genomic data and the need for methods to evaluate genomic variants. The majority of variants have unclassified phenotypic consequences, and their systematic exploration is complicated by data resources that are not easily obtainable or combinable. There is a need for more effective, user-friendly genome analysis tools that include interdisciplinary annotations and resources to suit the needs of both novices and bioinformatics experts. Rapid identification of somatic variants relevant to the progression and treatment of cancer are of particular importance to facilitate timely precision patient care. Maintaining patient privacy and data security places additional constraints on variant annotation and analysis, and requires systems that do not expose protected data.

Highly informative variant and gene characteristics are distributed across thousands of published works, spanning resources from the medical, biologic, and bioinformatics domains, including experimental assays, computational variant effect prediction, evolutionary context, population databases, and established pharmacologic relevance. This abundance of variant and gene annotations challenges researchers to broadly discover and deploy the best resources, as well as incorporate them within custom annotation pipelines. Furthermore, prediction algorithm software often requires nontrivial computational expertise to install, configure, and run. Recently, genome-wide precomputation of predictor outputs for every possible input variant has been undertaken to make computational tools more accessible. Databases that host precomputes, such as dbNSFP (database for nonsynonymous single-nucleotide polymorphisms’ functional predictions),^2,3^ have been instrumental in exposing users to new tools. However, the datasets available from these resources were designed for machine rather than human access and require substantial programming investment before a user can incorporate them into an annotation pipeline.

Decision support framework (DSF) software tools have been created to integrate multiple annotation resources.^4^ Well-designed DSFs require substantial software development; therefore, the majority of DSFs are not freely available. The remaining minority of DSFs are either Web-based portals that expose private data or downloadable tools with complicated installation and configuration requirements.^5-7^ One such Web-based DSF is the Cancer-Related Analysis of Variants Toolkit^8^(CRAVAT), which prioritizes somatic mutations.^9^ In this work, we present OpenCRAVAT, an extension of CRAVAT with improved data security, a much larger collection of annotations, and the capability to generate dynamic and customizable pipelines.

OpenCRAVAT is a freely available open-source framework for the annotation and visualization of human genetic variation and genomic elements. The framework can rapidly generate publication-quality visualizations of gene networks, provide the distribution of variants per protein, and support BAM file visualization with an embedded version of the Integrative Genomics Viewer (IGV).^10^ Designed to comprehensively annotate both well-characterized and novel somatic and germline variation, the framework can be flexibly adapted to suit a wide spectrum of human variation research projects. In this article, we describe the underlying architecture and present several case studies.

## METHODS

### Framework Architecture

OpenCRAVAT is written in Python, and all code is stored on a public repository. It is open source and free of charge to users, with both command-line and graphical user interface (GUI) functionality. OpenCRAVAT can be installed via user-friendly wizard or through pip. The framework is built around 2 main components: a base module and a store where users can download additional modules. Modules include input format converters, gene mappers, annotators, output format reporters, and graphical widgets. The base module includes converters that support Variant Call Format (VCF), tab-delimited (TSV), and comma-delimited (CSV) text files; a mapper that projects genome positions to transcript; protein sequence and protein structure coordinates; a set of basic widgets and reporters that generate output results files in sqlite3, Excel, TSV, CSV, and VCF formats. OpenCRAVAT supports GRCh38, GRCh37, and GRCh36 human genome reference assemblies, and variants are mapped to all GENCODE isoforms.^11^ The store offers a large selection of modules, including additional installable converters (Ancestry, 23andMe, dbSNP identifiers); annotators for somatic, de novo, and germline variation (coding and noncoding); and associated widgets and reporters (VCF, pipeline-friendly TSV and CSV).

The store is available through both GUI and command-line interface. Within the GUI, available modules are displayed in a format similar to an app store, where each tool is represented by a tile containing documentation, update status, and one-click installation. After installation, OpenCRAVAT downloads each resource locally, which enables secure analysis of private data. The open store is built for continuous community-driven development, so that newly developed tools and resources can be uploaded and made available to a wide audience. Addition of new resources to the store requires data descriptions, appropriately formatted annotation data, and a small script to allow incorporation of the data by OpenCRAVAT. Module developers can select whether to openly publish their data or restrict access, with the option to share the module directly with collaborators.

### Using OpenCRAVAT

**FIG 1.**
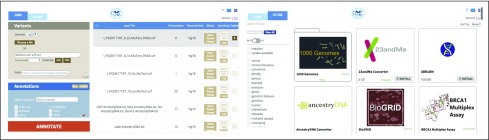
Screenshot of the OpenCRAVAT (Open Custom Ranked Analysis of Variants Toolkit) graphical user interface submission and store pages.

Configurable workflows within OpenCRAVAT can be created and executed in either the command line or GUI. OpenCRAVAT generates annotations for input files of human genetic variants. VCF, annotated VCF, basic tabular file format, dbSNP identifiers, 23andMe, and Ancestry.com files are supported. To accommodate family and cohort studies, multiple VCF files can be selected and merged within a single annotation run, in addition to support for multisample VCF files. For each annotation run, the user has the option to include all installed annotators or a subset, allowing for the creation of custom annotation pipelines (Fig 1). On completion of a run, the interactive results viewer can be used for exploratory data analysis and filtering.

**FIG 2.**
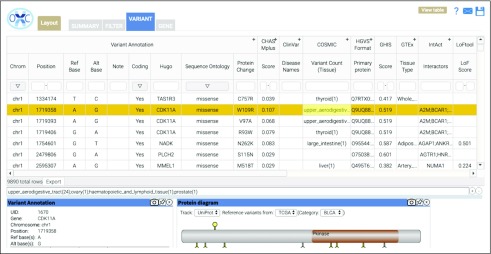
Screenshot of the OpenCRAVAT (Open Custom Ranked Analysis of Variants Toolkit) graphical user interface variant analysis table.

Accessible via both command line and GUI, the viewer comprises 4 tabs: Summary, Variant, Gene, and Filter (Fig 2). The Summary tab displays graphical representation of the submitted variant characteristics, as well as submission details, including the selected annotations and data source versions. The Variant and Gene tabs are divided into an interactive table and widget pane. The interactive table displays each variant or gene on a particular row along with the corresponding user-selected annotations. The widget pane includes several interactive elements which graphically display additional information and visualizations of the annotators, including the IGV with BAM file support and a Protein Diagram to visualize protein-level variation. Within the viewer, table columns and widgets can be resized or hidden, and layout preferences can be saved, shared, and applied to other annotation runs. The Filter tab allows users to generate and save filters, which identify variants in selected samples or genes, population allele frequency ranges, genomic locations, by sequence ontology, or custom annotator-specific thresholds. For example, after installation of the gnomAD module, users may choose to annotate their sample with gnomAD allele frequency and then use the Filter tab to reduce their analysis to variants with allele frequency < 0.01. For more complex filtering tasks, the Query Builder allows users to build advanced SQL queries on the Filter tab of the interactive result viewer.

OpenCRAVAT can be installed locally on a user’s computer or on a server, allowing multiple users to submit annotation runs on the same system, with administrator monitoring and maintenance. The server implementation adds user authentication, user-specific storage, user access to history, and shared access to analysis and visualization results. Server installation can be performed on both a shared local system or in a cloud environment, where results storage can be controlled and protected data are secure. The entire catalog of resources can be stored in one place and shared among many users, in addition to analysis results.

## RESULTS

In the following case studies, we illustrate the capacity of OpenCRAVAT to evaluate phenotypically relevant genetic variations within inputs of differing size and composition.

### Case Study 1: Variant Prioritization in Multiple Lesion Cancer Samples

Among the somatic variants present in a tumor, a small number of mutations are believed to “drive” tumor growth and may be useful for diagnosis, prognosis, patient stratification, clinical trial eligibility, and selection of appropriate therapies. Of particular interest are clonal driver mutations that occurred in the initiating tumor cell and are present in all tumor cells. Identification of these originating mutations can be enhanced by evaluating mutations from multiple tumor biopsies, including precursor lesions, primary cancers, and metastases from a single patient.

In this case study, we investigated early candidate driver mutations in a patient with high-grade serous ovarian cancer (CGOV62), using VCF and BAM files from a published genomic study of high-grade serous ovarian cancers, including fallopian tube precursor lesions; fallopian tube and ovarian tumors; and omental, rectal, and appendiceal metastases; with a normal fallopian tube epithelium control sample.^12^ BAM files from whole-exome sequencing were downloaded from the European Bioinformatics Institute (EGAS00001002589), and VCF files were generated with MuTect v.1.1.7 using default parameters.^13^

**FIG 3.**
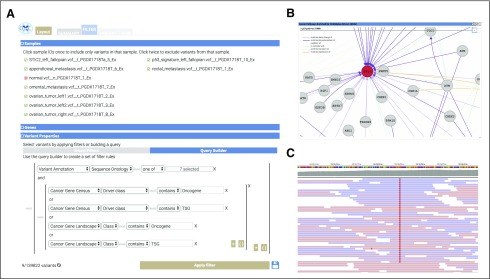
Components of the OpenCRAVAT (Open Custom Ranked Analysis of Variants Toolkit) graphical user interface used in Case Study 1. (A) The Query Builder filters applied to identify potential cancer driver mutations. (B) NDEx network enriched for mutations within these samples. (C) Screenshot of Integrative Genomics Viewer reads for a tumor sample.

The analysis was carried out using the Query Builder (Fig 3A) by:

1. Installing cancer-related annotation modules (Cancer Gene Census^14^ and Cancer Gene Landscapes^15^), computational predictors (CHASMplus OV^16^ and MutPred^17^), and a visualization module (IGV).
2. Within the interactive interface, selecting the genome version used in the study (hg18), uploading VCF files for each biopsied lesion, selecting the annotators listed in the step 1, and clicking the Annotate button.
3. On the Filter tab, filtering by sample to exclude any germline variants that were present in the normal fallopian tube epithelium sample.
4. To focus on loss-of-function mutations in tumor suppressor genes (TSG) and missense mutations in oncogenes (OG), applying a Sequence Ontology–filter to select either (missense, splice site, frameshift and nonframeshift indels, and stop gain) for TSG or (missense and nonframeshift indels) for OG.
5. Retaining mutations within known OG and TSG as provided by either the Cancer Gene Landscapes or Cancer Gene Census.

Nine mutations were retained after applying these filters, of which 2 were likely clonal mutations: *RANBP2*:p.M933I and *TP53*:p.T126N. These mutations were observed in seven of the eight lesions. In the original study, the *TP53* mutation was found in an eighth lesion by deep targeted sequencing. The *TP53* mutation is a known driver, with CHASMplus OV *P* value < .01 and is predicted by MutPred to result in loss of sheet structure (*P* = .0457). The NDEx widget was used to explore interaction partners of the mutated proteins, and the NDEx enrichment tool identified 13 *TP53*-associated networks from the National Cancer Institute Pathway Interaction Database^18^ (Fig 3B). For each truncal mutation, the normal and tumor BAM files were loaded into IGV for viewing and manual validation (Fig 3C). Manual inspection verified that the mutation was truly somatic, it was not present in normal tissue (data not shown), and there was no apparent strand bias.

### Case Study 2: Identifying Driver Missense Mutations Among Metastases

We analyzed exome and genome sequencing data for 76 untreated metastases from 20 patients with breast, colorectal, endometrial, gastric, lung, melanoma, pancreatic, and prostate cancers from a recent study on the heterogeneity of functional driver mutations in cancer metastases.^19^ This analysis was performed by:

1. Installing the CHASMplus annotator to score mutations as likely cancer drivers and tsvreporter to generate simple tab-delimited output.
2. Assembling a tab-delimited file of 15,765 somatic mutations identified in the study by Reiter et al^19^.
3. Using the command-line interface to generate a CHASMplus score for each mutation: cravat reiter_et_al_2018.txt -n Reiter_2018 -t tsv -l hg19–cleanup -d output.
4. Running a python script *fdr.py* that took in the output file and created a qvalue for each mutation, which is a correction of the CHASMplus *P* value for multiple hypothesis testing using the false discovery rate of 0.05.

**FIG 4.**
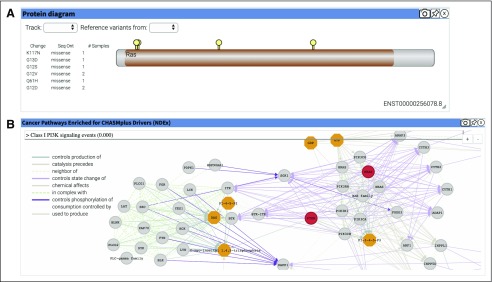
Visualization widgets that describe the variants analyzed in Case Study 2. (A) Protein Diagram displaying the 6 protein-coding *KRAS* variants. The Ras Pfam^29^ domain is indicated in tan along the Protein Diagram. (B) NDEx graphical widget showing the “Class I PI3K signaling events” network, where the mutation-harboring *KRAS* and *PTEN* are shown in red.

In total, 56 mutations were predicted as drivers, with a significant qvalue (q < 0.01). These included well-known oncogenic alleles (*KRAS*:p.G12D, *SMAD4*:p.D351G, and *PTEN*:p.R173H^20^). There are 6 *KRAS* mutations present in these samples, including two mutations that have been observed in more than a single sample (*KRAS*:p.G12D and *KRAS*:p.G12V; Fig 4A). The NDEx widget shows that the *KRAS* and *PTEN* variants both affect the “Class I PI3K signaling events” network (Fig 4B). All data and code needed to replicate the analysis are available at the OpenCRAVAT website^21^.

### Case Study 3: Clinically Actionable Germline Variants in an Individual Genome

We identified germline variants that are suspected to be relevant to cancer in a phenotypically normal individual obtained from the Personal Genome Project (Profile hu3BDC4B).^22,23^ For this analysis, we used databases of single-nucleotide variations (SNVs), indels, and genes with relevance to cancer, including hereditary predisposition: ClinVar,^24^ PharmGKB,^25^ and the ClinGen Gene annotator, which includes gene-disease associations curated by the ClinGen consortium.^26^ The findings for each annotator are as follows:

1. The ClinVar annotator identified dozens of variants relevant to cancer. Variants with the highest potential for clinical relevance include a variant that is protective for lung cancer, two risk-factor variants (lung cancer and cutaneous malignant melanoma), 5 pathogenic noncoding SNVs (acute myeloid leukemia [AML] with maturation), a pathogenic intronic SNV in *EHBP1* associated with hereditary prostate cancer, and 16 drug-response variants that affect the dosage, efficacy, toxicity/adverse drug reaction or response to various cancer drugs.
2. The ClinGen Gene annotator identified variants within 43 genes related to cancer phenotypes. Of these, the most impactful variant was a frameshift deletion in *PALB2*, which ClinGen has identified to be related to “familial ovarian cancer; hereditary nonpolyposis colon cancer; hereditary breast carcinoma; Fanconi anemia complementation group.” An additional 25 genes related to breast, ovarian, colon, and colorectal cancers are affected by missense variants.
3. The PharmGKB annotator identified two variants. First, an intronic variant in *GLDC* was associated with increased response to citalopram and escitalopram in people with major depressive disorder. *GLDC* had been annotated by the ClinGen Gene module as associated with glycine encephalopathy. Second, a 3 prime UTR variant of *ENOSF1* was associated with response to methotrexate.

The majority of variants in this patient have no known clinical relevance. Among the variants highlighted by the ClinVar module, only a single variant, related to hereditary prostate cancer, may be suitable to consider informing the patient to encourage early intervention. The ClinGen Gene module does not appear to be of clinical utility for this patient, with the potential exception of the frameshift deletion affecting *PALB2*, which has been associated with susceptibility to several cancer types. If the individual receives pharmacologic treatment of cancer in their lifetime, the variant-drug annotations from PharmGKB and ClinVar may have clinical utility.

### Case Study 4: Occurrence of Somatic Mutations Within Molecular Subgroups Among 182 Patients With AML

For genetically heterogenous cancer types such as AML, partitioning patients into clinical subgroups based on their genomic alterations carries significant prognostic implications. Individuals with AML have previously been partitioned into 11 genomic subgroups, based on patterns of comutation.^27,28^ In this case study, we assessed the prevalence of these clinical subgroups using somatic mutations from 182 patients with AML, sequenced by The Cancer Genome Atlas and obtained from the Genomic Data Commons (gdc.cancer.gov). Genomic subgroups that were defined by inversions, translocations, and gene fusion events were omitted from this analysis because these variant types are not currently supported by OpenCRAVAT:

1. **Genomic subgroup 1:** AML with *NPM1* mutation. We identified 22 patients in this subgroup, with a total of 11 *NPM1* mutations, of which 2 were observed in more than one sample (*NPM1*:p.W288Cfs*12 and *NPM1*:p.V156E).
2. **Genomic subgroup 2:** AML with mutated chromatin, *RNA*-splicing genes, or both. For the second subgroup, we identified 13 patients. The selection of genes under consideration were derived from the list of genes that are required to harbor at least one driver mutation for classification in this subgroup: *RUNX1*, *ASXL1*, *BCOR*, *STAG2*, *EZH2*, *SRSF2*, *SF3B1*, *U2AF1*, *ZRSR2*, or *MLL*^PTD^. A total of 17 protein-coding mutations affect these genes, of which three were observed in more than one sample (*STAG2*:p.T958S *STAG2*:p.L957F, *SF3B1*:p.L833F).
3. **Genomic subgroup 3:** AML with *TP53* mutations, chromosomal aneuploidy, or both. We identified 37 patients in this subgroup, with a total of nineteen *P53* mutations, of which five were observed in more than one sample (*TP53*:p.S378P, *TP53*:p.T377P, *TP53*:p.A70G, *TP53*:p.T231P, *TP53*:p.R248Q).
4. **Genomic subgroup 4:** AML with biallelic *CEBPA* mutations. No patients exhibited biallelic *CEBPA* mutations. However, 5 patients had a single mutation (*CEBPA*:p.V308dup, *CEBPA*:p.R300C, *CEBPA*:p.R343Afs*79, *CEBPA*:p.R286Pfs*35, *CEBPA*:p.T310_Q311insKWNP).
5. **Genomic subgroup 5:** AML with *IDH2* R172 mutations and no other class-defining lesions. We identified 4 patients in this subgroup with *IDH2*:p.R172K mutations. No other patients had a mutation that affected R172.

Of the 182 total patients in this cohort, 76 were assigned into the 5 molecular subgroups based on protein-coding somatic mutations. The remaining 50 patients most likely harbored inversions, translocations, and/or gene fusion events. We observed that 11 of the total 49 mutations occurred in more than 1 patient and may reflect recurrent driver mutations with prognostic value.

## DISCUSSION

OpenCRAVAT is a flexible and dynamic system to annotate, evaluate, and visualize the characteristics of genetic variation. It has been designed to enable rapid characterization of variants, including functional impact, pharmacologic annotations, and both known and predicted relevance of genetic variants to disease, including cancer. The open store contains dozens of resources relevant to variant interpretation, with new additions weekly. Selection of specialized converters, annotators, and filtering criteria enable researchers to carry out complex analyses and integrate information from a wider array of resources than previously possible.

We have described a framework that includes both an advanced GUI for biologists and a command-line interface that supports advanced use cases, including development of custom bioinformatics pipelines. Both GUI and command line can be leveraged in the cloud to handle processing of genomes from large patient populations. Finally, because the OpenCRAVAT store is designed to be community driven, we have incorporated more than 100 tools from dozens of universities and institutes in the past year, and we are actively recruiting tool and resource developers.
